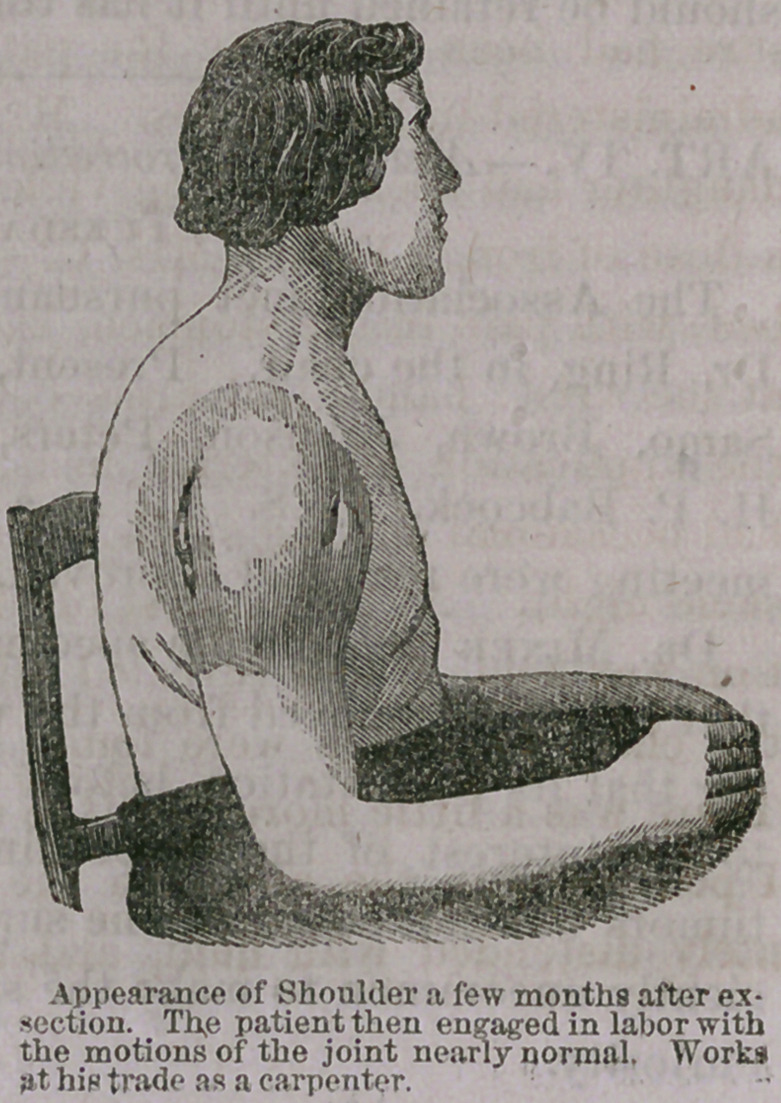# Abstract of Proceedings of Buffalo Medical Association

**Published:** 1865-10

**Authors:** Joseph A. Peters

**Affiliations:** Sec’y.


### ART. IV.—*Abstract of Proceedings of Buffalo Medical Association.*

TUESDAY EVENING, September 5, 1865.

The Association met pursuant to adjournment, the President, Dr. Ring, in the chair. Present, Drs. Miner, Hauenstein, Strong, Samo, Brown, Johnson, Peters, and Gleason, members, and Dr. H. P. Babcock, U. S. N., as a guest. The minutes of the last meeting were read and approved.

DR. MINER presented a specimen of bony tumor which he had that morning removed from the vicinity of the shoulder-joint, hoping that its presentation before the Society would add somewhat to the interest of the proceedings. Possibly the growth of such tumors was not as rare as he supposed, but certainly it was sufficiently uncommon to make the specimen of interest, if not of rare curiosity.

About two years since he had made exsection of the head of the humerus of a patient aged 48 years, carpenter by trade, who had suffered from immense enlargement of the shoulder-joint, which was found to be caused by some disease of the bones composing the joint. After this operation the patient had fully recovered the use of the arm and had worked with acceptable ability at his trade of house carpenter. Within the last few months, the shoulder had again commenced to enlarge, and great accumulations of feebly organized fibrin mixed with serum, had formed round the joint, and within the mass could be detected hard, slightly movable tumors. Upon opening down upon them they were found to be growths of bone which had formed between the glenoid cavity and the exsected end of the humerus. There were six or eight in number, their size varying from a quarter of an inch to an inch and a half in diameter. The bone structure proper was enveloped in a cartilaginous covering which appeared as if it was itself being transposed into bone—was the first material deposited out of which the bone was later formed. These tumors were unattached to other bones, were independent bony growths.—There was not great pain, but the strength of the arm was lost and the disability from this cause and the great size it had attained was complete.

The case has before been reported, soon after the exsection was made, and the following cuts were used to show the appearances both before the exsection and a few months later. They are again inserted as giving a better idea of the appearance than can be done by words.

Outline of Shoulder before first operation.

Appearance of Shoulder a few months after exsection. The patient then engaged in labor with the motions of the joint nearly normal. Works at his trade as a carpenter.

Dr. Gleason presented the following account of the poisoning of Mrs. Crocker:

I was called to see Mrs. C. on the 16th of August; found upon my arrival that Dr. Tobey had also been called, and arriving before I did, had prescribed. I was requested to see the patient, which I did; found her suffering greatly; extremeties cold; pulse small and rapid; head hot; countenance expressing great anxiety; abdomen tender on pressure; complained of great pain in the back and stomach; very thirsty; very restless, with frequent and violent vomiting. I advised Mr. C. to send immediately for Dr. Rochester, as he had usually employed him in the absence of Dr. Eastman, his family physician.

I was invited by Mr. C. to partake of a lunch with himself and daughter. It being past my dinner hour, I accepted the invitation, eating a little lamb chop, a cracker, and drinking a cup and a half of coffee. In about ten minutes after eating, I began to feel nauseated; started immediately for my office, promising, at the request of Mr. C. to call and notify Dr. Rochester that he was wanted immediately. Before reaching Dr. R.'s office I was taken with violent vomiting. This occurred about half an hour after eating. I was informed, late in the afternoon, that Dr. R. had not gone, and could not go, and according to the request of Mr. C., in case Dr. R. could not come, I returned, taking with me the hydrated sesqui-oxide of iron, as an antidote for arsenic, which I was sure had been taken by the patient, as well as myself, which I administered in large doses. Mr. C. informed me that he and his daughter had been vomiting violently in the afternoon. Gave him a dose of iron. Found Mrs. C. still very restless, tossing about in bed, with pain in the stomach, back and head; pulse imperceptible at the wrist; hands and arms cold; lower extremities warm, from the application of external heat; great thirst and great anxiety; had occasional sinking spells. Patient died about 12 o'clock of the same night. At the request of the friends a *post mortem* examination was held, which revealed slight *rigor mortis.* Upon opening the chest the lungs were found in a perfectly healthy condition. There was a little more than the usual amount of pericardial fluid. Upon opening the abdomen the stomach was found to be inordiately distended with fluid, and upon attempting its removal, although no force was used, the duodenum was ruptured. A ligature was immediately applied; the stomach was also ruptured slightly, to which a ligature was also applied, and its contents preserved. A portion of the illium, spleen, liver, pancreas and duodenum were removed and delivered to Prof. Hadley for examination.

Prof. H. informs me that he has as yet found no arsenic in the contents of the stomach, but has found it in the blood from the liver. The mesenteric glands were enlarged. Upon opening the stomach there was found a great quantity of brownish fluid. The mucus coat was slightly injected, softened and puffy with slight abrasions.

Dr. Peters gave an account of a post-mortem in a case of stone, of which he promised a more extended account when he could hear from the attending physician.

Dr. Strong wished to relate an extraordinary case of tolerance of opium in a child. Was called in July to see a child fourteen months old that was suffering from diarrhœa. The little one had always been of a fretful disposition, and had been largely dosed with opiate cordials. He soon found that ordinary doses of opium exercised no control over the disease, or the accompanying pain, and was obliged to increase the dose of laudanum to 10 or 11 drops, before any effect was produced, and even then no sleep was induced. The disease was somewhat controlled, but a relapse followed with cholaric discharges, and he had finally to give 20 drops of laudanum every four hours. With this treatment the child got better, and he discontinued attendance.

About the middle of August the child was taken sick again with the same disease, and he found that opiates having been given freely, *ad interim*, he had to increase the dose to 25 drops, and finally to half a teaspoonful of laudanum. He tried an injection of half a teaspoonful of laudanum with starch, but it was not retained, and he finally gave in an enæma three-fourths of a grain of morphia, which controlled the discharges. In all this time there was very little hypnotic effect produced, the child sleeping but little, the pupils very slightly contracted, and little or no effect produced on the brain. Digestion was not at all impaired by the opium. Believed the child to be laboring under mesenteric disease, and to that and the habit of giving cordials alluded to, the extraordinary tolerance of opium was undoubtedly due.

DR. RING thought a great impetus had been given to the use of opium by certain distinguished physicians, and he feared with ill effect. Thought its prolonged use likely to produce effusion of the brain.

DR. SAMO was led by experience to differ from some of the gentlemen in regard to the use of opium. He used it a great deal, especially with children, and had never had any occasion to regret it. Uses chiefly Dovers powder or paregoric. Has treated many cases of dysentery with Dovers powders alone. Did not believe in the necessity of calomel in bowel complaints. Spoke also of the extreme prevalence of the habit of opium-eating, and thought its sale should be better regulated by law.

DR. BABCOCK asked Dr. Samo if he had ever tried ipecac in the treatment of dysentery, and bore testimony to its value.

Diarrhœa, dysentery, cholera morbus, hooping cough, and typhoid fever were reported as prevailing, and the Association adjourned. JOSEPH A. PETERS, Sec'y.

## Figures and Tables

**Figure f1:**
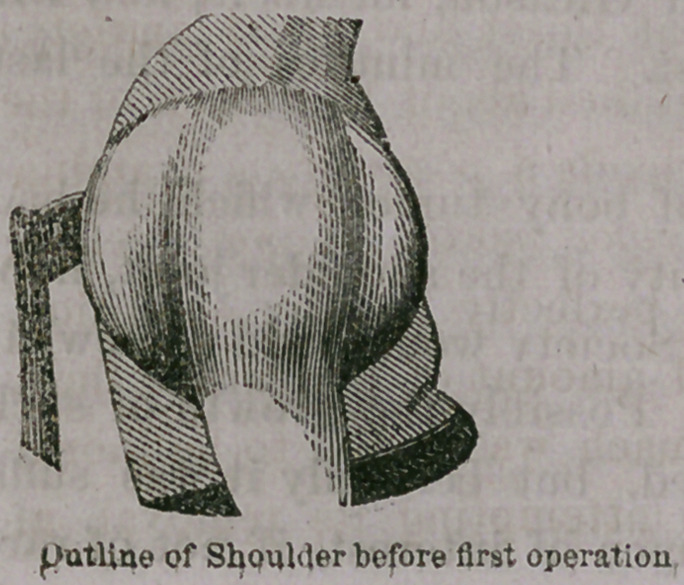


**Figure f2:**